# Erythropoietin Interacts with Specific S100 Proteins

**DOI:** 10.3390/biom12010120

**Published:** 2022-01-12

**Authors:** Alexey S. Kazakov, Evgenia I. Deryusheva, Andrey S. Sokolov, Maria E. Permyakova, Ekaterina A. Litus, Victoria A. Rastrygina, Vladimir N. Uversky, Eugene A. Permyakov, Sergei E. Permyakov

**Affiliations:** 1 Institute for Biological Instrumentation, Pushchino Scientific Center for Biological Research of the Russian Academy of Sciences, Pushchino, 142290 Moscow, Russia; fenixfly@yandex.ru (A.S.K.); janed1986@ya.ru (E.I.D.); 212sok@gmail.com (A.S.S.); mperm1977@gmail.com (M.E.P.); ealitus@gmail.com (E.A.L.); certusfides@gmail.com (V.A.R.); permyakov.s@gmail.com (S.E.P.); 2 Department of Molecular Medicine and Byrd Alzheimer’s Research Institute, Morsani College of Medicine, University of South Florida, Tampa, FL 33612, USA; vuversky@usf.edu

**Keywords:** erythropoietin, S100, protein-protein interactions, four-helical cytokine

## Abstract

Erythropoietin (EPO) is a clinically significant four-helical cytokine, exhibiting erythropoietic, cytoprotective, immunomodulatory, and cancer-promoting activities. Despite vast knowledge on its signaling pathways and physiological effects, extracellular factors regulating EPO activity remain underexplored. Here we show by surface plasmon resonance spectroscopy, that among eighteen members of Ca^2+^-binding proteins of the S100 protein family studied, only S100A2, S100A6 and S100P proteins specifically recognize EPO with equilibrium dissociation constants ranging from 81 nM to 0.5 µM. The interactions occur exclusively under calcium excess. Bioinformatics analysis showed that the EPO-S100 interactions could be relevant to progression of neoplastic diseases, including cancer, and other diseases. The detailed knowledge of distinct physiological effects of the EPO-S100 interactions could favor development of more efficient clinical implications of EPO. Summing up our data with previous findings, we conclude that S100 proteins are potentially able to directly affect functional activities of specific members of all families of four-helical cytokines, and cytokines of other structural superfamilies.

## 1. Introduction

Erythropoietin (EPO) is a pleiotropic monomeric glycosylated short-chain four-helical cytokine (SCOP entry 4000852; newly synthesized EPO is the 193-residue long protein containing a 27-residue long signal peptide, which is removed upon maturation, giving rise to 166 residues in mature form, 30.4 kDa), mainly produced by adult kidney type I interstitial cells, fetal liver hepatocytes, and Ito cells [1,2,3,4,5]. EPO signals through homodimer of the EPO receptor (EPOR), the heterodimer of the EPOR and the β common receptor (CD131, βcR) or the Ephrin type-B receptor 4 (EphB4) [6,7,8,9]. EPO stimulates basal and stress-induced erythropoiesis (in the case of bleeding, hypoxia, etc.) via binding to the EPOR on the erythroid progenitor surface, thereby triggering STAT5, Ras/MAPK and PI3K/Akt pathways, which drive expression of the genes promoting cell survival, proliferation and maturation, along with feedback inhibition of the EPOR signaling [6]. EPO affects multipotent mesenchymal stem cells, which leads to bone remodeling, induction of angiogenesis and secretion of trophic factors [3]. It also serves a tissue-protective role upon binding to the EPOR and the βcR receptors (co-expressed upon tissue injury in kidney, liver, heart and nervous system), which activates STAT3, MAPK and PI3K/Akt pathways, leading to immunosuppression and inhibition of apoptosis, inflammation and fibrosis [7]. Several cytoprotective effects of EPO are ascribed for the brain, kidney, heart, lung and retina, including anti-apoptotic, anti-oxidative, anti-inflammatory, anti-fibrotic, and pro-angiogenic activities, and regulation of endoplasmic reticulum stress [10]. EPO is shown to modulate activity of various cells of the innate and adaptive immunity [11]. Finally, EPO signaling through EphB4 was reported to promote cancer progression via STAT3 signaling [8].

Recombinant EPO and its derivatives are widely clinically used for treatment of anemia due to chronic kidney disease, rheumatoid arthritis, myelosuppressive therapy for cancer, myelodysplasia, zidovudine in HIV-infected patients, therapy for chronic hepatitis C, in the case of preterm anemia, and for minimization of blood transfusions in patients undergoing surgery, etc. [12]. The global EPO drugs market size has already reached 12 billion USD and is estimated to grow at a compound annual growth rate (CAGR) of 10%, according to the Market Data Forecast. Other EPO-based therapeutic applications are under development, including prevention of dementia, Alzheimer’s disease, Parkinson’s disease, schizophrenia, bipolar disorder, depression, ischemic stroke, traumatic brain injury, diabetes mellitus, acute and chronic lung diseases, various ocular diseases, severe COVID-19, as well as EPO use in the regenerative medicine [1,3,10,13,14,15,16,17,18,19,20,21]. However, the clinical use of the EPO-based medicines is limited due to the pleiotropic nature of this cytokine, leading to serious adverse reactions, such as arterial hypertension, cerebral convulsion/hypertensive encephalopathy, thrombo-embolism, iron deficiency, development of pure red cell aplasia, and the possibility of cancer progression [8,22,23]. Hence, the factors affecting EPO functioning need detailed exploration.

Despite its wide physiological and pathological implications, the interactions of EPO with extracellular soluble proteins have not been reported to date, to the best of our knowledge. In the present work, we show that under the in vitro conditions, EPO recognizes specific members of the multifunctional S100 protein family, which contains over 20 regulatory Ca^2+^-binding proteins of the EF-hand superfamily [24,25,26]. S100 proteins consist of the two Ca^2+^-binding motifs, linked by a ‘hinge’ region: a low-affinity pseudo EF-hand (α-helices I and II), and a canonical EF-hand (α-helices III and IV) [27]. S100 proteins exhibit wide cell/tissue-specific expression, localize in cytosol/nucleus/extracellular space, and interact with numerous partners (receptors, ion channels, cytoskeletal proteins, enzymes, transcription factors, nucleic and fatty acids), regulating the pathways governing cell differentiation/proliferation/death, energy metabolism, metal homeostasis, inflammation, pathogen resistance, etc. [24,25]. Some of the S100 proteins are released into the extracellular space, followed by their binding to various cell surface receptors: RAGE, TLR4, ErbB1 (EGFR), ErbB3, ErbB4, CD36, CD68, CD147, neuroplastin-β, CD166, 5-HT_1B_, IL-10R and SIRL-1 [25,28,29]. Thereby, S100 proteins affect cellular signaling in an autocrine/paracrine manner as damage-associated molecular patterns or ‘alarmins’. Furthermore, some of the extracellular S100 proteins interact with cytokines, including specific members of interleukin 6 (‘IL-6’) family [30,31,32,33], IFN-β [28,34], IL1α/FGF1 [35,36], FGF2 [37] and EGFR ligands [38]. Both intra- and extracellular actions of S100 proteins mediate their involvement into wide range of oncological, cardiovascular, respiratory, neurological, inflammatory and autoimmune diseases, which promotes their use in diagnostics and therapy [25,26,39,40,41,42]. The EPO-S100 interactions revealed here uncover a higher complexity level in regulation of EPO-mediated processes, which may be significant for development of novel therapeutic strategies.

## 2. Materials and Methods

### 2.1. Materials

Human erythropoietin produced in CHO cells (Epoetin β; glycoprotein comprising 165 residues and lacking a signal peptide) was from «PHARMAPARK» LLC (Moscow, Russia). Human S100A1/A4/A6/A7/A8/A9/A10/A11/A12/A13/ A14/A15/B/P proteins and the Y88R mutant of the protein S100P were prepared in *E. coli* according to previously published studies [30,32,34]. Recombinant human calmodulin (‘CaM’) was purified according to ref. [43]. Usp2 was prepared as described in [44]. Restriction enzymes were from Thermo Scientific^TM^ (Waltham, MA, USA) Hen egg white lysozyme was from Sigma-Aldrich Co. (St. Louis, MO, USA) Protein concentrations were measured spectrophotometrically using extinction coefficients at 280 nm calculated according to [45] (see Supplementary materials, Table S1).

Human *S100A2/A3/A5/A16* genes encoding proteins S100A2/A3/A5/A16 (UniProt entries P29034, P33764, P33763, Q96FQ6, respectively) were obtained from the DNASU Plasmid Repository (https://dnasu.org/DNASU/Home.do) (accessed on 10 January 2020): clones HsCD00504608, HsCD00504655, HsCD00821358, HsCD00504657, respectively.

HEPES, phosphate, Tris, imidazole, DTT, glycerol, sodium chloride, and sodium hydroxide were from PanReac AppliChem. Tricine, IPTG and PMSF were purchased from Helicon (Moscow, Russia). CaCl_2_, EDTA and TWEEN 20 were from Sigma-Aldrich Co. TOYOPEARL^®^ SuperQ-650M resin was purchased from Tosoh Bioscience (Tokyo, Japan). HiPrep™ 26/60 Sephacryl^®^ S-100 HR column was from GE Healthcare. Bio-Scale™ Mini Profinity™ IMAC cartridges, ProteOn™ GLH sensor chip and amine coupling kit were from Bio-Rad Laboratories, Inc. (Hercules, CA, USA).

### 2.2. Construction of Plasmids

Human *S100A2/A3/A5/A16* genes were cloned into pHUE (Histidine-tagged Ubiquitin Expression) vector [46] between SacII and HindIII restriction sites.

### 2.3. Expression and Purification of S100A2/A3/A5/A16 Proteins

Samples of recombinant human S100A2/A3/A5/A16 proteins were prepared as follows. Cells of *E. coli* BL21 (DE3) containing pLacIRARE plasmid were transformed with the pHUE-S100 plasmid. The cells were grown at 37 °C in 1 L of 2YT medium with 100 µg/mL ampicillin, shaking at 200 rpm, until the optical density at 600 nm reached 1 AU. Expression of the ubiquitin-S100 chimera was induced by 0.5 mM IPTG. The cells were grown at 25 °C for 4 h, harvested by centrifugation at 5000× *g* for 15 min at 4 °C, resuspended in 30 mL of lysis buffer (50 mM phosphate, 5 mM imidazole, 1 mM PMSF, 1 mM 2-ME, 1 M NaCl, 0.1% TWEEN 20, 0.1 mg/mL hen egg white lysozyme, pH 8.0), and disintegrated using a French press (IBI RAS, Russia). The lysate was centrifuged at 25,000× *g* for 40 min at 4 °C. The supernatant was loaded onto 5 mL Bio-Scale™ Mini Profinity™ IMAC Ni-charged column. The resin was washed with 50 mL of the lysis buffer. The protein was eluted with 10 mM phosphate, 300 mM imidazole, 1 mM 2-ME, 150 mM NaCl, pH 7.5 buffer. The fractions containing ubiquitin-S100 chimera were joined, dialyzed at 4 °C against 20 mM Tris-HCl pH 8.2, 1 mM DTT (buffer A), and treated with USP2 ubiquitin-specific protease (50–100-fold molar excess of the chimera over the enzyme, 16 h at 37 °C), and after that loaded onto a 6 mL TOYOPEARL^®^ SuperQ-650M anion exchange column and washed with the buffer A. The first peak has been discarded. The S100 protein was eluted by a linear gradient of NaCl (0–1.5 M) in the buffer A (50 mL; flow rate of 1 mL/min). The collected S100 protein was further purified using a HiPrep™ 26/60 Sephacryl^®^ S-100 HR gel filtration column equilibrated with PBS (flow rate of 1 mL/min). The purified protein was dialyzed at 4 °C against 1:1 (*v*/*v*) PBS-glycerol mixture and stored at −20 °C.

### 2.4. SPR Studies

Surface plasmon resonance (SPR) measurements of EPO affinity to S100 proteins were performed at 25 °C mainly as described earlier [28], using ProteOn™ XPR36 spectrometer (Bio-Rad Laboratories, Inc., Hercules, CA, USA). Ligand (0.05 μg/mL EPO) was immobilized by amine coupling on ProteOn™ GLH sensor chip surface (up to 3 000 resonance units, RUs). Analyte (0.25–4 μM S100A1/A2/A3/A4/A5/A6/A7/A8/A9/A10/A11/A12/A13/A14/A15/A16/ B/P(wild-type and Y88R)/CaM) in 10 mM HEPES, 150 mM NaCl, 0.05% TWEEN 20, pH 7.4 buffer with 1 mM CaCl_2_ or 1 mM EDTA was passed over the chip, followed by its flushing with the buffer. The ligand was regenerated by application of 20 mM EDTA solution, pH 8.0. The double-referenced SPR sensograms were analyzed using a heterogeneous ligand model, assuming presence of two populations of a ligand (L_1_ and L_2_) that bind an analyte molecule (A):

(1) $$L_{1}+A\begin{array}{c} \begin{array}{c} \;k_{a1} \end{array}\\ ⇄\\ \begin{array}{c} \;k_{d1}\\ \;K_{d1} \end{array} \end{array}L_{1}A\;L_{2}+A\begin{array}{c} \begin{array}{c} \;k_{a2} \end{array}\\ ⇄\\ \begin{array}{c} \;k_{d2}\\ \;K_{d2} \end{array} \end{array}{\;L}_{2}A\;$$

where *k_a_* and *k_d_* refer to kinetic association and dissociation constants, respectively; *K_d1_* and *K_d2_* are equilibrium dissociation constants: *K_d_* = *k_d_*/*k_a_*. The equilibrium and kinetic dissociation/association constants were evaluated using Bio-Rad ProteOn Manager™ v.3.1 software (Hercules, CA, USA) for each analyte concentration, followed by averaging of the resulting values (*n* = 5).

### 2.5. Structural Characterization of S100P Protein

Buffer conditions: pH 7.4, 10 mM tricine-KOH, 1 mM EDTA/CaCl_2_. Calcium depletion of wild-type and Y88R S100P was performed according to ref. [47]. Thermal stabilities of apo-forms (10–20 μM) of the proteins were estimated from thermal denaturation experiments monitored by tyrosine fluorescence as previously described [31]. Quantitative estimates of the protein (9–10 μM) secondary structure contents at 20 °C were performed by far-UV circular dichroism (CD) using CDPro software (Colorado State University, Fort Collins, CO, USA) according to ref. [48]. Crosslinking of the proteins (0.7 mg/mL) with 0.02% glutaric aldehyde was performed at 20 °C for 16 h, followed by SDS-PAGE, staining with Coomassie Brilliant Blue R-250 and the analysis described in ref. [48].

### 2.6. Modeling of EPO-S100 Complexes

The models of tertiary structures of EPO-S100 A2/A6/P complexes were built using ClusPro docking server [49], based on the structures of human EPO (PDB [50] entry 1BUY, NMR) and Ca^2+^-loaded dimers of human S100A2 (chains A, B of 4DUQ, X-ray), S100A6 (chains A, B of 1K9K, X-ray) and S100P (chains B, D of 2MJW, NMR). The balanced scoring scheme was used for calculations of the interaction energies. Ten most populated clusters of the complex structures (numbered from 0 to 9) were selected. The center of each cluster represents a putative model of the complex. Distributions of the contact residues in the docking models over the protein sequences were calculated using Python 3.3 programming language (implemented in PyCharm v.3.0.2 development environment), Matplotlib Python plotting library and NumPy numerical mathematics extension. The residues included into five or more docking models were considered as the most probable residues of the binding site. The models most closely covering the residues of the probable binding sites were taken for the illustration. The tertiary structure models were drawn with molecular graphics system PyMOL v.1.6.9.0 [51]. The numbering of the contact residues is according to the PDB entries.

### 2.7. Search of Diseases Associated with EPO and S100 Proteins

The data on diseases associated with EPO (UniProt ID P01588) and either S100A2 (UniProt ID P29034), S100A6 (UniProt ID P06703) or S100P (UniProt ID P25815) were collected from the human disease databases DisGeNET v7.0 [52] and Open Targets Platform v.20.09 [53] as described in ref. [28]. The DisGeNET entries were manually curated; false positive records were removed.

## 3. Results and Discussion

### 3.1. Conformation-Dependent Interaction between EPO and Specific S100 Proteins

**Figure 1:**
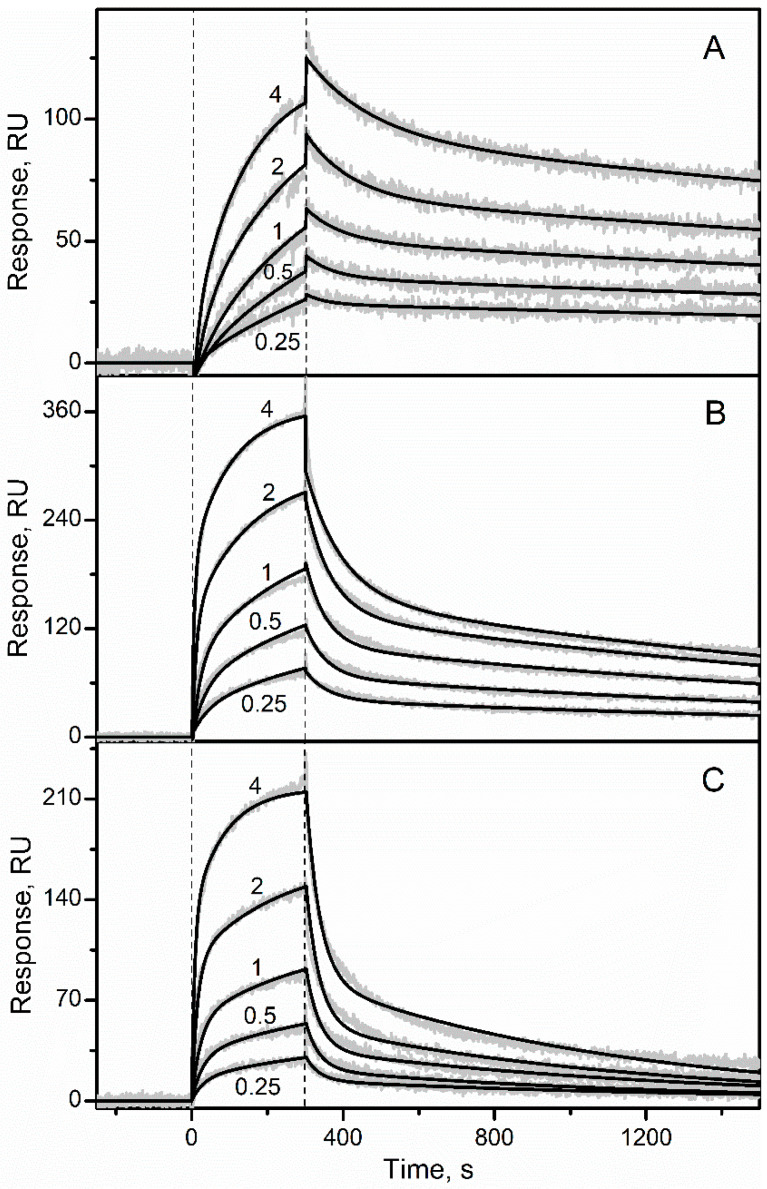
Kinetics of the interaction between EPO and Ca^2+^-loaded S100 proteins at 25 °C (pH 7.4, 1 mM CaCl_2_), followed by SPR spectroscopy using EPO as a ligand and S100A2 (panel (**A**)), S100A6 (**B**) or S100P (**C**) as an analyte. Molar concentrations of the analyte (µM) are indicated. The grey curves are experimental, while the black curves are theoretical, calculated according to the heterogeneous ligand model (1) (see Table 1).

Recombinant glycosylated human EPO was immobilized on the surface of SPR sensor chip by amine coupling and solutions of the recombinant human S100A1/A2/A3/A4/A5/A6/A7/A8/A9/A10/A11/A12/A13/A14/A15/A16/B/P/CaM were passed over the chip at 25 °C, using the pH 7.4 buffer containing 1 mM CaCl_2_. While no effects were observed for the Ca^2+^-loaded S100A1/A3/A4/A5/A7/A8/A9/A10/A11/A12/ A13/A14/A15/A16/B/CaM at a concentration of 1 µM (Figure S1), the SPR sensograms for Ca^2+^-bound S100A2/A6/P proteins (0.25–4 µM) exhibited the analyte concentration-dependent effects (Figure 1 and Figure S1). The dissociation phase of the sensograms reveals two distinct dissociation processes: (1) a relatively fast process with half-life time (t_1/2_) of 60 s; (2) a much slower process with t_1/2_ value exceeding 1000 s. The heterogeneous ligand model was previously shown to be well suited for description of S100-cytokine interactions [28,30,32,33,34]. Therefore, the kinetic SPR data were described by the heterogeneous ligand model (1) (Figure 1 and Figure S2) with the lowest equilibrium dissociation constants, *K_d_*, ranging from 81 nM to 0.5 µM (Table 1). Meanwhile, Ca^2+^-free (1 mM EDTA) forms of the S100 proteins at a concentration of 1 µM did not interact with EPO, as revealed by the lack of the SPR effects (data not shown).

As EPO is linked to the surface of the SPR chip via amino groups, it is possible that EPO can be exposed to the analyte in several preferential conformations, differing in their ability to recognize the analyte. Some of the EPO conformations may preclude the interaction with the analyte due to sterical hindrance, while others are characterized by an “open” conformation. The latter conformations could correspond to different analyte-binding sites or different conformations of the same site.

Overall, EPO recognized three S100 proteins (S100A2/A6/P) of the eighteen S100 proteins studied in a conformation-dependent manner, with a strict preference for the Ca^2+^-loaded conformers of the S100 proteins. This phenomenon was probably due to the Ca^2+^-induced solvent exposure of hydrophobic residues of the S100 proteins, promoting their target recognition [27,54,55,56]. Importantly, the S100 proteins specific to EPO belong to the category of ‘promiscuous’ S100 proteins, able to bind several ligands with a high affinity [57]. Meanwhile, the other ‘promiscuous’ S100 proteins (S100A1/A3/A4/A5/B [57]) turned out to be non-specific to EPO, indicating incomplete cross-reactivity among representatives of the ‘promiscuous’ S100 proteins. It should be emphasized, that the S100A2/A6/P proteins are evolutionary fairly distant from each other.

In fact, the pairwise sequence identities between them, calculated using UCSF Chimera software [58] and MUSCLE algorithm as implemented in EMBL-EBI service [59], range from 48.9% (S100A2–S100A6) to 34.4% (S100A6–S100P). For this reason, the revealed EPO-S100 interactions are non-redundant.

Although full-length EPO contains a signal peptide, the example of CLCF1, which has a signal peptide, but needs association with CRLF1 for efficient secretion [60], indicates that the interactions between EPO and S100 proteins (also lack signal peptides) could favor secretion of both interaction partners. Alternatively, S100 binding to EPO could serve as a mechanism for regulation of the activity of this cytokine. The regulatory role of S100A1/A4/P proteins was previously shown for another four-helical cytokine, interferon β (‘IFN-β’) [28,34]: IFN-β-induced suppression of viability of MCF-7 breast cancer cells is inhibited by the S100 proteins. The potential ability of S100 proteins to affect EPO activity is especially valuable, considering that EPO interactions with extracellular soluble proteins have not been reported to date.

As equilibrium homodimer dissociation constants for the Ca^2+^-loaded S100A6/P do not exceed 0.5 µM [34,61], the SPR estimates of their affinities to EPO (Table 1), measured at the S100 concentrations from 0.25 µM to 4 µM (Figure 1), correspond to dimeric states of S100A6/P. Meanwhile, conversion of S100A2/A6/P proteins into monomeric form should promote their interaction with EPO. For instance, affinity of four-helical cytokine interleukin 11 (‘IL-11’) to monomeric state of S100P (K_d_ of 1 nM [31]) exceeds that to dimeric S100P [30] by 1.5 orders of magnitude. Furthermore, monomerization of S100A1/A4/A6/P proteins increases their affinities to IFN-β by at least two orders of magnitude [28,34]. Indeed, basal serum S100P level (1 nM [62]) is much lower than its homodimer dissociation constant (64 nM [61]), suggesting monomeric state of S100P in serum. In this case, *K_d_* values for the EPO-S100 interactions may approach the elevated blood levels of S100A2/A6/P proteins, observed under pathological conditions: 1.4 nM for S100A2 [63] and 3–5 nM for S100A6/P [62,64]. Furthermore, local concentrations of EPO and extracellular S100 proteins in damaged tissues producing these proteins are expected to be considerably higher, compared to those in blood, thereby further favoring their interaction.

As elevated blood EPO concentrations under some pathological conditions (22.8 IU/L = 6 pM [65]) are much below the basal blood level of S100A2/A6/P proteins (0.4 nM–1 nM [62,63,64]), EPO is unable to modify signaling of the extracellular S100 proteins via their receptors. Instead, the S100A2/A6/P binding could affect EPO signaling.

### 3.2. Modeling of EPO-S100 Protein Complexes

**Figure 2:**
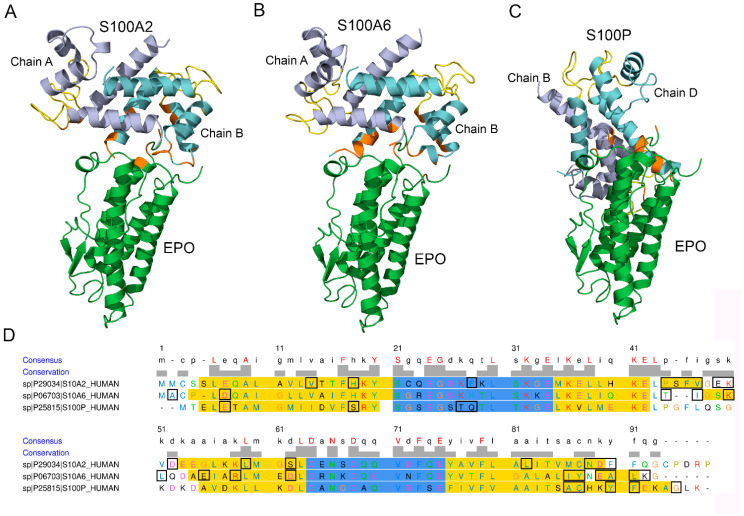
Panels (**A**–**C**) the models of tertiary structures of EPO (shown in green) complexes with the Ca^2+^-bound S100A2/A6/P dimers. Structures of EPO and the S100A2/A6/P proteins (PDB entries 1BUY, 4DUQ, 1K9K and 2MJW, respectively) are used for the modelling by the ClusPro docking server [49]. The Ca^2+^-binding loops are shown in yellow. The contact residues are orange-colored. Panel (**D**) mapping of the contact residues of the S100A2/A6/P proteins (shown in rectangles) onto their aligned amino acid sequence (MUSCLE algorithm as implemented in EMBL-EBI service [59]). The Ca^2+^-binding loops and helical regions according to the PDB entries are marked in blue and yellow, respectively. The residue letters are colored using the Clustal X color scheme. The scheme is prepared using the UCSF Chimera molecular modelling system [58].

Modeling of the tertiary structure of the complex between EPO and a Ca^2+^-loaded S100A2 dimer using the ClusPro docking server [49] predicts (Figure 2A,D) that S100A2 chain A interacts with EPO via helix II (P44, S45, F46, V47), ‘hinge’ region (E49 and K50), helix III (D52, L59 and S62) and helix IV (L82, N88, D89, F90). Chain B of the dimer binds EPO via helix I (V14, H18) and residue F28 of the pseudo EF-loop. The S100A2-binding region of EPO includes residues of the C-terminal helix (E159, R162), and the C-terminal residue R166.

The analogous modeling of structure of the EPO complex with the Ca^2+^-loaded S100A6 dimer shows (Figure 2B,D) that the S100A6 chain A binds EPO through ‘hinge’ region (T43, I44, K47, L48), helix III (E52, R55 and D59), helix IV (I83, Y84, E86, A87, L88), and the C-terminal residue K89. The S100A6 chain B interacts with EPO via the N-terminal residues A2 and D6. The S100A6-binding region of EPO includes residues of the N-terminus (P2, R4, D8), R10 of the N-terminal helix, and the C-terminal residue R162. The same residue K89 of S100A6 was previously shown to bind the V domain of RAGE (PDB entry 2M1K).

The modeling of the complex between EPO and the Ca^2+^-loaded S100P dimer predicts (Figure 2C,D) that the S100P chain B interacts with EPO via helix IV (A84, C85, Y88, F89, G93), while chain D of the dimer binds EPO via M1, helix I (E5, S16) and pseudo EF-loop (T25, Q26). The respective S100P-binding region of EPO contains residue K52 of the loop between α-helices I and II, and the C-terminal residue R166. The same residues E5, C85, Y88, F89 and G93 of S100P were previously shown to bind the V domain of RAGE (PDB entry 2MJW).

Notably, EPO is predicted to interact with S100A2/A6/P proteins via only 2–5 residues, which is an unexpectedly low number of contact residues and could be a consequence of the rigid body approximation used in the modelling. Meanwhile, some of the predicted contact residues of EPO (D8, R10 and K52—bind to S100A6 and S100P, respectively) are involved into interaction with extracellular domain of EPOR (PDB entry 1EER), which points out that the S100 binding to EPO could interfere with its signaling via EPOR. Similarly, S100A1/A4/P binding to IFN-β inhibits signaling of this cytokine [28,34]. Nevertheless, the absence of noticeable cellular effects for S100P and S100A6 association with IL-11 [30] and IFN-β [34], respectively, shows that S100 binding does not necessarily affects signaling of the helical cytokines. Furthermore, S100A4 binding-induced enhancement of amphiregulin-mediated signaling via EGFR⁄ ErbB2 [38] demonstrates that S100 association with a receptor ligand can even stimulate signaling of the latter.

Although orientations of S100A2 and S100A6 molecules relative to the EPO molecule within the predicted EPO-S100 complexes are very similar, the S100P protein exhibits notably different orientation (Figure 2A–C). The analogous phenomenon was previously predicted for S100A1/A4/A6/P binding to IFN-β [34]. Thus, S100 binding to the helical cytokines suggests variability in mutual orientations of the interacting partners. Nevertheless, the S100A2/A6/P proteins are predicted to interact with the same apical region of the EPO molecule. The proximity of Ca^2+^-binding loops of one of the S100A2/A6/P chains to the contact surface in the model structures (Figure 2A–C) could favor Ca^2+^ sensitivity of the S100 - EPO interactions, in accord with the SPR data.

Mapping of the S100A2/A6/P residues predicted to bind EPO onto the aligned amino acid sequence of the S100 proteins shows (Figure 2D) that they share only residue 90 of the aligned sequence. Notably, this residue was implicated in the S100A1/A4/P binding to IFN-β [34]. To probe the significance of the residue 90 of the aligned sequence for EPO recognition, we have replaced it in the S100P protein (Y88) by Arg. The resulting Y88R mutant did not reveal noticeable affinity to EPO in SPR measurements using Y88R (1 µM) as an analyte (Figure S2C), thereby arguing contribution of S100P residue Y88 into the process of EPO binding. Meanwhile, despite preservation of a quaternary structure of Y88R mutant, as evidenced by crosslinking with glutaric aldehyde (data not shown), it exhibits a decline in α-helicity according to far-UV CD data (57% versus 64–67% for wild-type S100P) and lowered thermal stability of its apo-form (mid-transition temperature estimated by intrinsic fluorescence of 63 °C versus 86 °C for the wild-type protein). The substantial structural consequences of the Y88R substitution prevent the conclusion that Y88 residue is involved in an interaction with EPO.

### 3.3. Intrinsic Disorder and Interactivity of EPO

**Figure 3:**
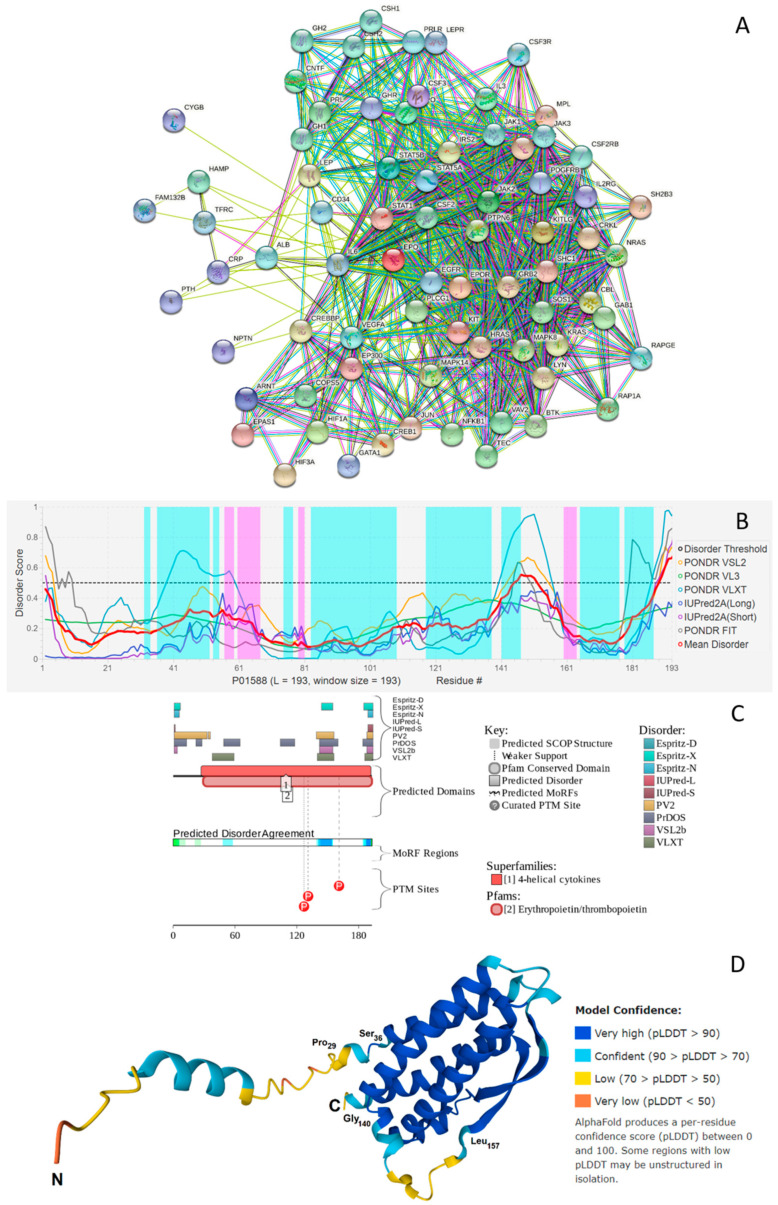
(**A**) STRING-based analysis of the interactivity of the human EPO (UniProtKB-P01588) using the high confidence level of 0.7. STRING generates a network of expected associations based on the predicted and experimentally-validated information on the interaction partners of a protein of interest [66]. In the corresponding network, the nodes correspond to proteins, whereas the edges show predicted or known functional associations. Seven types of evidence are used to build the corresponding network, and are indicated by the differently colored lines: a green line represents neighborhood evidence; a red line—the presence of fusion evidence; a purple line—experimental evidence; a blue line—co-occurrence evidence; a light blue line—database evidence; a yellow line—text mining evidence; and a black line—co-expression evidence [66]. (**B**) Per-residue disorder profile of human EPO generated by the DiSpi web crawler that aggregates the results from PONDR^®^ VLXT [67], PONDR^®^ VL3 [68], PONDR^®^ VLS2B [69], PONDR^®^ FIT [70], IUPred2 (Short) and IUPred2 (Long) [71,72]. A threshold of ≥0.5 is used to identify disordered residues and regions in query proteins. Positions of α-helices and β-strands are shown as light cyan and pink areas, respectively. (**C**) Functional disorder profile generated for human EPO by the D^2^P^2^ platform (http://d2p2.pro/) (accessed on 29 December 2021) [73], which uses outputs of IUPred [71], PONDR^®^ VLXT [67], PrDOS [74], PONDR^®^ VSL2 [68,69], PV2 [73], and ESpritz [75] to generate disorder profile, where nine colored bars represent the location of disordered regions as predicted by these different disorder predictors. The blue-green-white bar in the middle of the D^2^P^2^ plot shows the predicted disorder agreement between nine disorder predictors, with blue and green parts corresponding to disordered regions by consensus. Above the disorder consensus bar are two lines with colored and numbered bars that show the positions of the predicted (mostly structured) SCOP domains [76,77] using the SUPERFAMILY predictor [78]. The red circles at the bottom of the plot show location of phosphorylation sites assigned using the outputs of the PhosphoSitePlus platform [79]. (**D**) Structure of the full-length human EPO modeled by AlphaFold [80].

Information accumulated so far on the various biological roles of EPO clearly emphasizes the immense multifunctionality of this protein. Most of these activities are somehow related to the ability of EPO to interact with multiple partners. This is illustrated by Figure 3A showing EPO-centered protein-protein interaction (PPI) network generated by the Search Tool for the Retrieval of Interacting Genes (STRING) platform [66]. This network includes 74 proteins engaged in 776 interactions thereby organizing a network with average node degree of 21 (i.e., on average, each protein there interacts with 21 partners). This network is characterized by the average local clustering coefficient of 0.66. Average local clustering coefficient defines how close the neighbors are to being a complete clique – if a local clustering coefficient is equal to 1, then every neighbor connected to a given node Ni is also connected to every other node within the neighborhood, and if it is equal to 0, then no node that is connected to a given node *N_i_* connects to any other node that is connected to *N_i_*. As the expected number of interactions in a similar size set of proteins randomly selected from the human proteome is equal to 174, this STRING-generated PPI network has significantly more interactions than expected, being characterized by a PPI enrichment *p*-value of <10^−16^. This observation indicates that the proteins in the EPO-centered PPI network have more interactions among themselves than what would be expected for a random set of proteins of similar size. Therefore, such an enrichment indicates that the proteins are at least partially biologically connected, as a group.

Analysis of this PPI network for the GO-based functional enrichment revealed that the most prominent biological processes in this network were Cellular response to chemical stimulus (GO:0070887; *p* = 1.64 × 10^−39^), Cell surface receptor signaling pathway (GO:0007166; *p* = 4.18 × 10^−38^), Cellular response to organic substance (GO:0071310; *p* = 2.32 × 10^−36^), Cellular response to cytokine stimulus (GO:0071345; *p* = 2.66 × 10^−33^), and Response to cytokine (GO:0034097; *p* = 2.76 × 10^−33^). The most common molecular functions were Protein binding (GO:0005515; *p* = 8.41 × 10^−19^), Signaling receptor binding (GO:0005102; *p* = 1.16 × 10^−17^), Cytokine receptor binding (GO:0005126; *p* = 4.16 × 10^−17^), Growth factor receptor binding (GO:0070851; *p* = 6.01 × 10^−13^), and Receptor ligand activity (GO:0048018; *p* = 3.97 × 10^−10^), whereas among the most common cellular components, one can find Receptor complex (GO:0043235; *p* = 5.28 × 10^−7^), Endosome lumen (GO:0031904; *p* = 1.16 × 10^−5^), Extracellular region (GO:0005576; *p* = 0.00015), Mast cell granule (GO:0042629; *p* = 0.0014), and Cell surface (GO:0009986; *p* = 0.0014).

Given multifunctionality and binding promiscuity is commonly associated with the presence of intrinsically disordered regions, next, we looked at the per-residue disorder predisposition of human EPO. Results of this analysis are represented in Figure 3B, which indicates the presence in this protein of several disordered and flexible regions. Here, protein region was considered ordered if it had an average disorder score (ADS) < 0.15. When 0.15 ≤ ADS < 0.5, the region was considered as moderately disordered or flexible, whereas ADS ≥ 0.5 correspond to the disordered protein region. As on average, the entire protein was characterized by the APS of 0.3 (as per the outputs of PONDR^®^ VSL2, which was selected based on its exceptional performance at the recent Critical Assessment of protein Intrinsic Disorder prediction (CAID) experiment [81], where it was ranked #3 of 43 methods evaluated on a dataset of 646 proteins from DisProt [82]), one can conclude that globally, EPO is a rather flexible protein. This is further conformed by the fact that based on its percent of predicted disordered residues (PPDR), EPO is classified as a moderately disordered protein. In this classification, two arbitrary cutoffs for the levels of intrinsic disorder are used to classify proteins as highly ordered (PPDR < 10%), moderately disordered (10% ≤ PPDR < 30%), and highly disordered (PPDR ≥ 30%) [83].

Complementary information on the intrinsic disorder predisposition of the full-length human EPO together with some important disorder-related functional information was retrieved from the D^2^P^2^ database (http://d2p2.pro/) (accessed on 29 December 2021) [73]. In agreement with Figure 3B showing the results of the multiparametric disorder analysis of this protein, Figure 3C illustrates that EPO is expected to have several disordered regions and includes three phosphorylation sites (Ser_127_, Ser_131_, and Thr_161_) surrounding the C-terminally located disordered region 139–159 (note, this region corresponds to residues 112–132 in the mature protein with deleted signaling peptide). It is tempting to hypothesize that the overall disorder status of this region is controlled by its phosphorylation status, and that this controlled disorder might be related to the modulation of protein functionality.

Further evidence of the presence of disordered and flexible regions in human EPO was retrieved based on the analysis of the structural propensity of this protein based on the modeling of its three-dimensional (3D) structure by AlphaFold2 [80], which is currently the most accurate computational methods to predict 3D protein structures from the protein sequence [84]. The use of this approach allowed visualization of the whole-length protein, including its signal peptide and regions of missing electron density in previously determined X-ray crystal structure of human EPO. In fact, in the structure of a complex of human EPO with extracellular domains of erythropoietin receptor (PDB ID: 1CN4), electron density is missing for residues 1, 124–130 (which actually overlaps with the 112–132 region predicted to be disordered, see above), and 163–166 [85]. Furthermore, this modeling provides a clue on the potential structural flexibility of a modeled protein, as the confidence of the AlphaFold structure prediction at the residue level is assumed to be a measure of the local flexibility of the polypeptide chain. In fact, it was pointed out in the study reporting the application of AlphaFold for the highly accurate protein structure prediction for the human proteome, that a considerable percentage of low-confidence residues in structures generated by this algorithm may be explained by some form of disorder, including both constitutively intrinsically disordered regions and regions that are structured only in complex with binding partners [86]. Figure 3D shows that in agreement with disorder predictions, EPO contains several flexible regions, such as the N-terminal tail and a 140–157 region, which is predicted to be disordered (see Figure 3B,C). Overall, it seems that the elongated left-handed four-helix bundle structure of EPO with the two β-strands in the crossover loops AB and CD is characterized by an asymmetric distribution of structural flexibility/disorder. In fact, Figure 3D shows that a side of the EPO four-helix bundle containing N- and C-termini is noticeably more flexible than the opposite site of the bundle. Importantly, this more disordered side of EPO is involved in interaction with its partners (including S100A2/A6/P and EPOR, see above), suggesting the importance of structural flexibility for binding promiscuity of this protein.

Altogether, these analyses revealed that EPO is a moderately disordered multifunctional protein with a capability to be engaged in promiscuous interactions with various proteins. The fact that in addition to the previously reported partners, EPO can bind the members of the S100 protein family in a conformation-depended manner further increases the functional repertoire of this important cytokine.

### 3.4. Human Diseases Associated to Dysregulation of EPO and S100 Proteins

To elucidate possible involvement of the EPO-S100 interactions into pathogenesis of human diseases, we searched the DisGeNET and Open Targets Platform (‘OTP’) databases for the diseases associated with simultaneous involvement of EPO and S100A2/A6/P.

DisGeNET contains 30 entries related to both EPO and S100A2: various neoplasms and periodontitis (Table S2). The OTP database includes 65 entries associated with both EPO and S100A2 (Table S3), but none of the entries had association scores exceeding 0.1.

DisGeNET contains 42 entries related to both EPO and S100A6: various neoplasms, acute myocardial infarction, myocardial ischemia, coronary arteriosclerosis, Alzheimer’s disease, amyloidosis, amyotrophic lateral sclerosis, and liver regeneration disorder (Table S4). The OTP database includes 171 entries associated with both EPO and S100A6 (Table S5). Consideration of the entries with association scores above 0.1 reveals neoplasms.

DisGeNET contains 4 entries related to both EPO and S100P: adenocarcinoma, ovarian neoplasm, polycystic ovary syndrome and prostatic neoplasms (Table S6). OTP database includes 84 entries associated with both EPO and S100P (Table S7). Consideration of the entries with association scores above 0.1 reveals neoplasms.

Examination of the DisGeNET database reveals that adenocarcinoma is simultaneously associated with EPO and S100A2, S100A6 and S100P. Meanwhile, the OTP database contains 30 entries related to these proteins (Table S8), but none of them had association scores above 0.1.

Overall, the bioinformatics analysis points out that the effect of S100A2/A6/P proteins on EPO activity could be relevant to development of neoplastic diseases, including cancer, and other diseases. The elevated levels of S100A2/A6/P in numerous cancers [40,87,88] may directly interfere with erythropoietic, cytoprotective, immunomodulatory and cancer-promoting activities of EPO.

## 4. Conclusions

In the present study, we have shown that the Ca^2+^-bound forms of some S100 proteins specifically interact under in vitro conditions with EPO, a short-chain four-helical cytokine. Hence, S100 proteins are potentially able to alter functional activities of specific members of all families of four-helical cytokines (SCOP ID: 3001717): short-chain cytokines (SCOP ID 4000852), long-chain cytokines (SCOP ID 400085; exemplified by specific cytokines of IL-6 family [30,31,32,33]), and interferons/interleukin-10 (IL-10) (SCOP ID 4000854; exemplified by IFN-β [28,34]). The structural basis and functional significance of the found EPO-S100 interactions remain to be explored, but the available data support a novel functional role of extracellular S100 proteins as modifiers of activity of specific four-helical cytokines, as well as representatives of cytokines of other structural superfamilies [35,36,37,38]. The revealed EPO-S100 interactions indicate a much higher complexity of regulation of EPO-mediated processes, likely contributing to diversity of functional activities of EPO. The detailed knowledge of distinct functional consequences of the EPO-S100 interactions could give us a clue to more efficient clinical implications of this highly important cytokine.

## Figures and Tables

**Table 1:** The parameters of EPO-S100 interaction at 25 °C, estimated from the SPR spectroscopy data (Figure 1) using the heterogeneous ligand model (1). *k_a_* and *k_d_* refer to kinetic association and dissociation constants, respectively; *K_d_*_1_ and *K_d_*_2_ are equilibrium dissociation constants: *K_d_* = *k_d_*/*k_a_*. The standard deviations are indicated.

Parameter\Analyte	S100A2	S100A6	S100P
*k_a_*_1_, M^−1^s^−1^	(2.2 ± 0.8) × 10^3^	(3.0 ± 0.6) × 10^3^	(2.0 ± 0.4) × 10^3^
*k_d_*_1_, s^−1^	(1.8 ± 0.4) × 10^−4^	(4.5 ± 0.5) × 10^−4^	(1.10 ± 0.11) × 10^−3^
*K_d_*_1_, M	(8.1 ± 2.2) × 10^−8^	(1.5 ± 0.3) × 10^−7^	(5.4 ± 1.2) × 10^−7^
*k_a_*_2_, M^−1^s^−1^	(1.0 ± 0.5) × 10^4^	(2.5 ± 0.7) × 10^4^	(1.6 ± 0.5) × 10^4^
*k_d_*_2_, s^−1^	(1.2 ± 0.5) × 10^−2^	(1.6 ± 0.3) × 10^−2^	(2.8 ± 0.4) × 10^−2^
*K_d_*_2_, M	(1.2 ± 0.7) × 10^−6^	(6.5 ± 2.6) × 10^−7^	(1.8 ± 0.5) × 10^−6^

## Data Availability

The data presented in this study are available in the article or supplementary material.

## Supplementary Materials

The following are available online at https://www.mdpi.com/article/10.3390/biom12010120/s1, Table S1. Molar extinction coefficients at 280 nm for the proteins used in the present study. Table S2. List of the human diseases associated with EPO and the S100A2 protein, according to the DisGeNET database. Table S3. List of the human diseases associated with EPO and the S100A2 protein, according to the OTP database. Table S4. List of the human diseases associated with EPO and the S100A6 protein, according to the DisGeNET database. Table S5. List of the human diseases associated with EPO and the S100A6 protein, according to the OTP database. Table S6. List of the human diseases associated with EPO and the S100P protein, according to the DisGeNET database. Table S7. List of the human diseases associated with EPO and the S100P protein, according to the OTP database. Table S8. List of the human diseases associated with EPO and S100A2, S100A6 and S100P proteins, according to the OTP database.

## Abbreviations

βcR: β common receptor (CD131); DTT, 1,4-dithiothreitol; EDTA, ethylenediaminetetraacetic acid; EphB4, Ephrin type-B receptor 4; EPO, erythropoietin; EPOR, erythropoietin receptor; HEPES, 4-(2-hydroxyethyl)piperazine-1-ethanesulfonic acid; PBS, phosphate-buffered saline; IFN-β, interferon β; IPTG, isopropyl-β-D-thiogalactoside; MAPK, mitogen-activated protein kinase; 2-ME, 2-mercaptoethanol; NMR, nuclear magnetic resonance; OTP, Open Targets Platform; PI3K, phosphatidylinositol-3-kinase; PMSF, phenylmethylsulfonyl fluoride; PPI, protein-protein interaction; RU, resonance unit; STAT, signal transducer and activator of transcription; SPR, surface plasmon resonance; Usp2, mouse ubiquitin specific peptidase 2; Tris, tris(hydroxymethyl)aminomethane.
