# Supplementary material for: Erythropoietin Interacts with Specific S100 Proteins
Source: Biomolecules. 2022 Jan 12;12(1):120. doi: 10.3390/biom12010120 (PMC8773746; doi:10.3390/biom12010120)
Supplement: Supplementary file 1 [file biomolecules-12-00120-s001.zip › biomolecules-1513431-supplementary.pdf]

Supplementary Materials

Erythropoietin interacts with specific S100 proteins

Alexey S. Kazakov[1], Evgenia I. Deryusheva[1], Andrey S. Sokolov[1], Maria E. Permyakova[1], Ekaterina A. Litus[1], Victoria A. Rastrygina[1], Vladimir N. Uversky[2], Eugene A. Permyakov[1,], and Sergei E. Permyakov[1]*

[1] Institute for Biological Instrumentation, Pushchino Scientific Center for Biological Research of the Russian Academy of Sciences, Pushchino, Moscow region, 142290 Russia
[2] Department of Molecular Medicine and Byrd Alzheimer's Research Institute, Morsani College of Medicine, University of South Florida, Tampa, FL, USA

***CORRESPONDING AUTHOR**: Dr. Eugene A. Permyakov, Institute for Biological Instrumentation, Pushchino Scientific Center for Biological Research of the Russian Academy of Sciences, Institutskaya str., 7, Pushchino, Moscow region, 142290 Russia; Email: epermyak@yandex.ru; Tel: +7 (495) 143-7741; Fax: +7(4967) 33 05 22

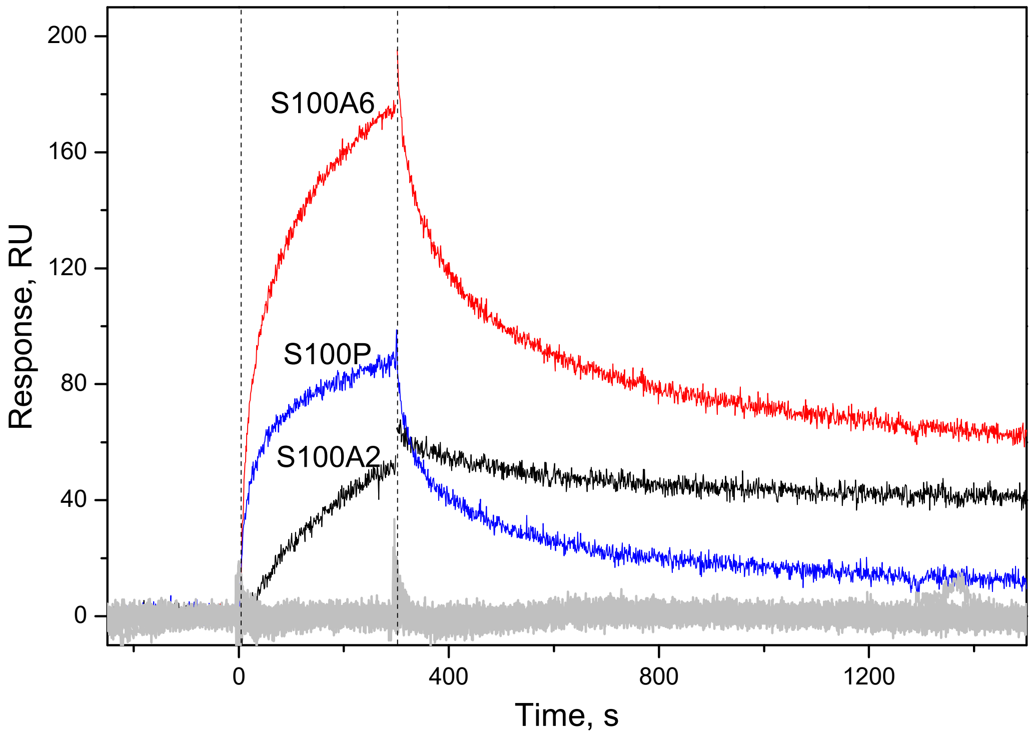

Figure S1. Kinetics of the interaction between EPO and Ca^{2+}-loaded S100 proteins at 25ºC (pH 7.4, 1 mM $CaCl_2$), followed by SPR spectroscopy using EPO as a ligand and S100 proteins/CaM (1 μM) as an analyte. The grey curves correspond to the data for S100A1/A3/A4/A5/A7/A8/A9/A10/A11/A12/A13/A14/A15/A16/B proteins and CaM.

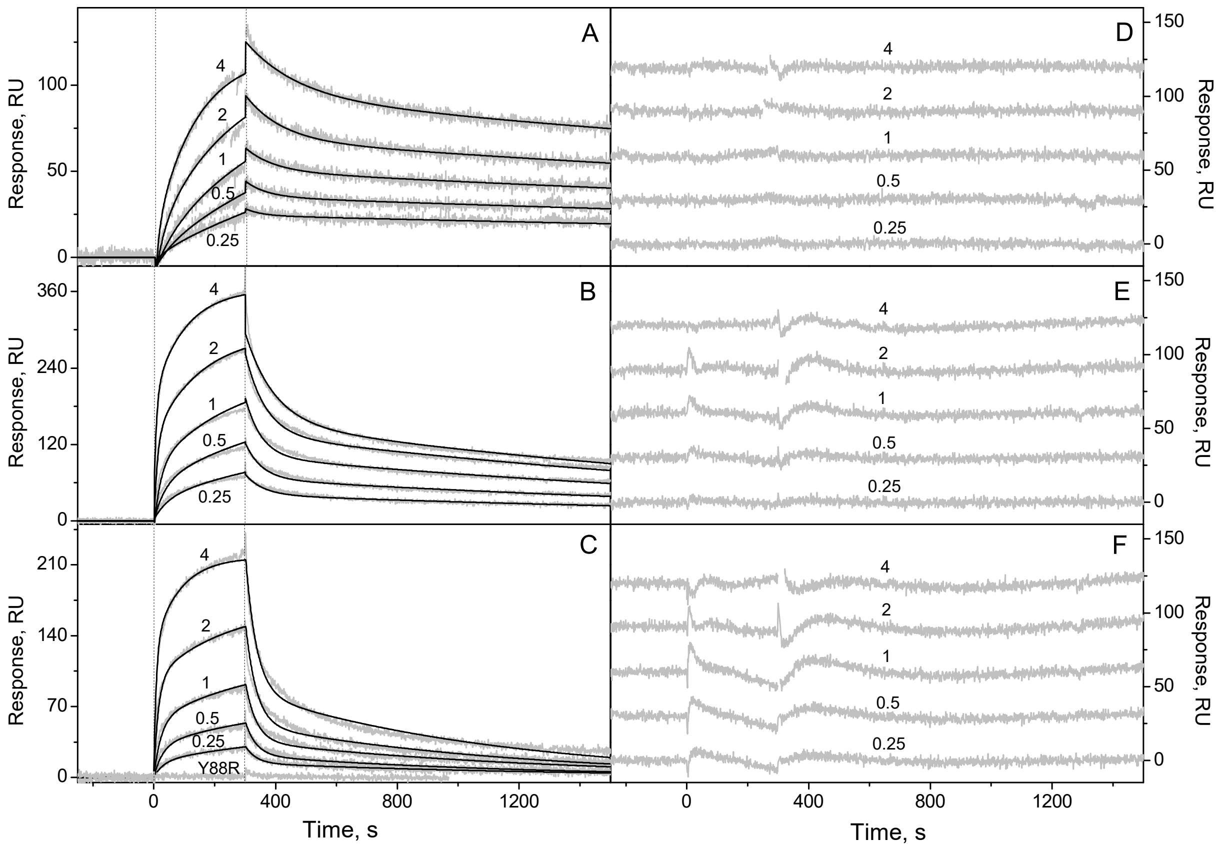

Figure S2. Kinetics of the interaction between EPO and Ca^{2+}-loaded S100 proteins at 25ºC (pH 7.4, 1 mM $CaCl_2$), followed by SPR spectroscopy using EPO as a ligand and S100A2 (panel **A**), S100A6 (**B**) or S100P (**C**) as an analyte. Molar concentrations of the analyte (μM) are indicated. The SPR data for kinetics of the interaction between S100P mutant Y88R (1 μM) and EPO are shown in panel **C**. The grey curves are experimental, while the black curves are theoretical, calculated according to the heterogeneous ligand model (1) (see Table 1). The panels **D**, **E** and **F** represent residuals of the fits for S100A2, S100A6 and S100P proteins, respectively.

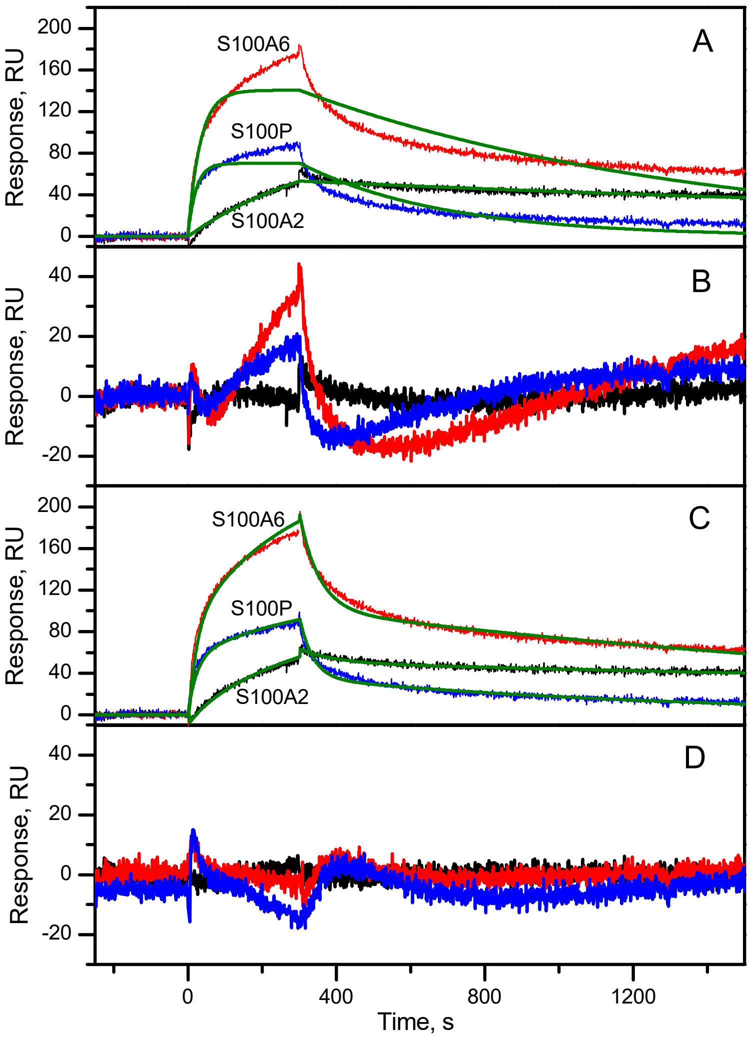

Figure S3. (**A**, **C**) Kinetics of the interaction between EPO and Ca^{2+}-loaded S100 proteins at 25ºC (pH 7.4, 1 mM $CaCl_2$), followed by SPR spectroscopy using EPO as a ligand and S100 proteins (1 µM) as an analyte. The black (S100A2), red (S100A6) and blue (S100P) curves are experimental, while the green curves are theoretical, calculated according to either 1:1 binding model (A) or the heterogeneous ligand model (1) (C). (**B**, **D**) Residuals of the fits for the 1:1 and heterogeneous ligand models, respectively. The color scheme is analogous to that used in panels A and C.

Table S1. Molar extinction coefficients at 280 nm for the proteins used in the present study, calculated according to ref. [1].

Protein	ε_{280nm}, $M^{-1}cm^{-1}$
EPO	22,710
S100A1	8,480
S100A2<br>S100A4<br>S100A10<br>S100A12<br>S100P<br>CaM	2,980
S100A3	14,440
S100A5<br>S100A6<br>S100A7<br>S100A11<br>S100A15	4,470
S100A8	11,460
S100A9<br>S100A13<br>S100A14	6,990
S100A16	12,950
S100B<br>Y88R S100P	1,490

Table S2. List of the human diseases associated with EPO and S100A2 protein, according to DisGeNET database (http://www.disgenet.org). PubMed identifiers of the references confirming the protein-disease associations are indicated.

№	*Disease*	*EPO*	*S100A2*
1	Adenocarcinoma	15709164	19238334; 17032501; 16232198; 15467767; 17032501; 27876462; 17940995; 16015609; 18656279;16367903; 11691825
2	Adult Ependymoma	14563949	18781180
3	Breast Carcinoma	21749867; 24502950; 31291309; 28247960; 20353997; 16207704; 21075308; 18084619; 29552165; 12118093; 30700319; 28418910; 24165569	11076656; 9481475
4	Carcinogenesis	16627979; 27145271; 24583149; 22843933; 12759237; 15769989; 16175858; 29552165	19725154; 11691825; 26916071; 17940995; 23337980
5	Carcinoma of lung	29345289	27876462; 18656279; 11691825; 24863947; 19118029
6	Childhood Ependymoma	14563949	18781180
7	Cholangiocarcinoma	23052842	25999659
8	Ependymoma	14563949	18781180
9	Head and Neck Carcinoma	15856028	17123307
10	Head and Neck Neoplasms	15671524	17123307
11	Lung Neoplasms	23861852	15467767; 17067748
12	Malignant Head and Neck Neoplasm	15856028	17123307
13	Malignant neoplasm of breast	16207704; 18084619; 12118093; 24502950; 21075308; 30700319; 28418910; 29552165; 24165569; 20353997; 31291309; 28247960; 28352153	9481475
14	Malignant neoplasm of lung	29345289	19118029; 18656279; 11691825; 27876462; 24863947
15	Malignant neoplasm of prostate	19567780; 30958616; 15467711; 29917167	16015609
16	Malignant Neoplasms	10916088; 21860424; 29719440; 28415825; 19264915; 16207704; 23830978; 29485009;	15800916; 23337980; 23996929; 11813862; 11076656

		8608241; 22814609; 31322257; 22843933; 18620752; 15467711; 20353997; 28985758; 31704854; 28629522; 27145271; 30971397; 31596520; 12667989; 20383466; 18431543; 31830663; 20384218; 24059228; 15591419; 26412593; 28504901; 17656101; 31828489; 29157782; 17253966; 28247960; 15856028; 23881169; 30969847; 25331796; 31691976; 30483799; 19536148; 18781955; 11325818; 31846977; 28795314; 1653276; 17038666; 30622244; 11950137; 12419827	
17	Neoplasm Metastasis	15856028; 29181060; 19536148; 16699298; 15856028; 28331341	19725154; 25874882; 25999659; 15800916; 9291441; 23996929; 19118029; 16273244; 16015609; 17123307; 17123307
18	Non-Small Cell Lung Carcinoma	29137269; 31752873; 16909043; 27494133; 15709164; 29345289; 29132013	27876462; 17689067; 15467767; 15313892; 18656279; 22033466; 24863947; 12400018; 19238334; 15800916; 19118029; 11691825
19	Pancreatic Ductal Adenocarcinoma	21829709	30570546
20	Periodontitis	29207066	21922197
21	Primary malignant neoplasm	22814609; 18781955; 30622244; 31704854; 30483799; 31322257; 28795314; 15856028; 19536148; 25331796; 26412593; 30969847; 31596520; 18620752; 16207704; 15467711; 19264915; 28629522; 22843933; 21860424; 29719440; 28247960; 17656101; 28415825; 29157782; 28504901;	15800916; 23996929; 11813862; 23337980

		29485009; 23881169; 17038666; 30971397; 11325818; 20353997; 12667989; 31828489; 31830663; 27145271; 15591419; 24059228; 31846977; 20383466; 23830978	
22	Prostate carcinoma	30958616; 29917167; 15467711; 19567780	16015609
23	Recurrent tumor	28587437	22833293
24	Secondary malignant neoplasm of lymph node	30854109; 17647284; 29181060; 15856028	19376121; 17123307; 27876462; 24318973
25	Squamous cell carcinoma	25182342; 15709164; 15967106; 16278379	18656279; 19238334; 16908593; 11304580; 17123307; 29453969; 21897749; 11956617
26	Squamous cell carcinoma of the head and neck	25182342; 15856028	17123307; 11304580
27	Stomach Carcinoma	30045276	24318973; 23337980; 12460893
28	Tumor Cell Invasion	20064603; 28247960; 22843933; 28629521; 25175278; 28795314; 15856028; 23028796; 24100272	16908593; 23996929; 16367903; 22747445; 25999659; 23337980; 24318973
29	Tumor Progression	23028796; 23305401; 19886790; 28415825; 26481148; 29491103; 26412593; 18781955; 24497137; 12759237; 17922127; 26575329; 19544471; 17429338; 28629521	22747445; 16367903; 17940995
30	Tumor Promotion	28703764	23996929

Table S3. List of the human diseases associated with EPO and S100A2 protein, according to Open Targets Platform database (https://www.opentargets.org), and corresponding association scores (https://docs.targetvalidation.org/getting-started/scoring).

№	*Disease*	*EPO*	*S100A2*
1	acne	0.002	0.008
2	acrokeratoelastoidosis of Costa	0.004	0.002
3	adenocarcinoma	0.008	0.012
4	adenoma	0.001	0.003
5	Alzheimer's disease	0.097	0.010
6	asthma	0.019	0.008
7	Barrett's esophagus	0.003	0.002
8	benign prostatic hyperplasia	0.002	0.027
9	bladder carcinoma	0.012	0.069
10	breast cancer	0.023	0.006
11	breast carcinoma	0.042	0.004
12	bronchopulmonary dysplasia	0.102	0.002
13	cancer	0.084	0.048
14	carcinoma	0.011	0.009
15	cervical carcinoma	0.012	0.002
16	cholangiocarcinoma	0.058	0.069
17	chromophobe renal cell carcinoma	0.002	0.012
18	clear cell renal carcinoma	0.001	0.039
19	colorectal adenocarcinoma	0.007	0.035
20	colorectal cancer	0.010	0.029
21	Crohn's disease	0.011	0.006
22	diabetes mellitus	0.094	0.002
23	familial adenomatous polyposis	0.011	0.004
24	gastric adenocarcinoma	0.013	0.015
25	gastric carcinoma	0.010	0.023
26	glioblastoma multiforme	0.026	0.012
27	head and neck squamous cell carcinoma	0.191	0.024
28	hepatocellular carcinoma	0.090	0.004
29	idiopathic pulmonary fibrosis	0.007	0.002
30	immature platelet fraction	0.003	0.001
31	in situ carcinoma	0.009	0.011
32	infection	0.092	0.003
33	invasive breast carcinoma	0.039	0.002
34	kidney neoplasm	0.026	0.004
35	laryngeal carcinoma	0.004	0.043
36	laryngeal squamous cell carcinoma	0.002	0.008
37	lung adenocarcinoma	0.009	0.017
38	lung carcinoma	0.008	0.002
39	lymph node metastatic carcinoma	0.007	0.003
40	medulloblastoma	0.004	0.043

41	melanoma	0.088	0.030
42	metastatic malignant neoplasm	0.004	0.002
43	neoplasm	0.112	0.061
44	non-small cell lung carcinoma	0.093	0.105
45	nonpapillary renal cell carcinoma	0.040	0.008
46	oral squamous cell carcinoma	0.001	0.004
47	osteosarcoma	0.052	0.003
48	ovarian carcinoma	0.013	0.009
49	pancreatic adenocarcinoma	0.017	0.008
50	pancreatic ductal adenocarcinoma	0.004	0.010
51	papillary renal cell carcinoma	0.012	0.009
52	periodontal disease	0.005	0.006
53	periodontitis	0.035	0.032
54	pes	0.039	0.002
55	platelet crit	0.033	0.023
56	psoriasis	0.001	0.029
57	pulmonary fibrosis	0.003	0.009
58	renal cell carcinoma	0.106	0.036
59	restless legs syndrome	0.007	0.004
60	sepsis	0.065	0.005
61	skin neoplasm	0.007	0.004
62	small cell carcinoma	0.002	0.004
63	squamous cell carcinoma	0.013	0.020
64	ulcerative colitis	0.013	0.009
65	Ulnar-mammary syndrome	0.001	0.001

Table S4. List of the human diseases associated with EPO and S100A6 protein, according to DisGeNET database (http://www.disgenet.org). PubMed identifiers of the references confirming the protein-disease associations are indicated.

№	*Disease*	*EPO*	*S100A6*
1	Acute myocardial infarction	22777420; 29752300; 28109258; 17896976; 31389586	28174168
2	Adenocarcinoma	15709164	12239456; 12239456; 10952782
3	Adult ependymoma	14563949	18781180
4	Alzheimer's disease	31763432; 28397428; 23662159; 31382012; 29743517; 27662300; 31453787; 31484803; 31199881; 30252487; 31493483; 31255922; 31660425; 22388478; 30656553	31440382; 15590066
5	Amyloidosis	18925459; 22388478	31440382
6	Amyotrophic lateral sclerosis	17439481; 17174305; 24820540; 17888545; 17142042; 30175978	11108968; 12152788; 15590066
7	B-cell lymphomas	28273832; 24394348	28123545
8	Breast carcinoma	24502950; 31291309; 28247960; 20353997; 16207704; 21075308; 18084619; 29552165; 12118093; 30700319; 28418910; 24165569	11267940; 25363551; 25454514; 28051137; 21459801; 22997041; 2448309; 11477119; 23029355; 9716033; 10517668; 23485561; 31809870; 26474308
9	Carcinogenesis	16627979; 27145271; 24583149; 22843933; 12759237; 15769989; 16175858; 29552165	15280928; 22295074; 28343163; 25760073; 22560296; 19891957; 20629554; 17970874; 25120023; 16340196
10	Carcinoma of lung	29345289	31497188; 16340196
11	Central neuroblastoma	31245372; 16500719; 12902921; 17045782; 12239177; 10087335; 15352172; 16407271	1998963; 7576953
12	Childhood ependymoma	14563949	18781180
13	Childhood neuroblastoma	16500719; 12902921; 17045782; 15352172; 12239177; 31245372; 16407271; 10087335	17579622; 1998963; 7576953; 19216736
14	Colon carcinoma	27903235; 29160911	29534068

15	Conventional (clear cell) renal cell carcinoma	15709172; 11986208; 19407488; 17640059; 17696210; 23305401; 21042786; 10916088; 16627979; 27468719; 28233444	25120023; 25760073
16	Coronary arteriosclerosis	28885393; 31778269; 31707794; 28315563; 25245553; 31741439; 17998053	28174168
17	Endometriosis	31262977; 12525458	26044826; 28075439; 29544367
18	Ependymoma	14563949	18781180
19	Glioblastoma	23070117; 30871240; 28629521; 17524506; 21749867; 9787178; 26178805; 24467192	19074870
20	Glioblastoma multiforme	24467192; 24379112; 21749867; 23070117; 26178805; 17524506; 28629521	19074870
21	Hepatoblastoma	30214643; 16175858; 9787178; 17218907	12859951
22	Liver carcinoma	8387202; 28587437; 12239177; 11408933; 1653242; 11592940; 24841514; 28739744; 11025195; 27827952; 9126339; 28743805; 23830978; 11090055; 8626614; 30723742; 24491563; 30367150; 2825172	24281831; 19048101
23	Liver regeneration disorder	31401982	12859951
24	Lung neoplasms	23861852	12859967
25	Malignant neoplasm of breast	16207704; 18084619; 12118093; 24502950; 21075308; 30700319; 28418910; 29552165; 24165569; 20353997; 31291309; 28247960; 28352153	25454514; 11267940; 21459801; 23029355; 28051137; 22997041; 10517668; 25363551; 2448309; 26474308; 25917868
26	Malignant neoplasm of lung	29345289	18620780

27	Malignant neoplasms	29719440; 28415825; 19264915; 16207704; 23830978; 29485009; 8608241; 22814609; 31322257; 22843933; 18620752; 15467711; 20353997; 28985758; 31704854; 28629522; 27145271; 30971397; 31596520; 12667989; 20383466; 18431543; 31830663	25120023; 29544367; 29552203; 19888321; 28123545; 18714402; 17970874; 15280928
28	Malignant squamous cell neoplasm	25182342	29053662
29	Malignant tumor of colon	29160911; 27903235	29053662
30	Melanoma	15743794; 21860424; 19536148	7654229; 9291441; 1737392; 28433799; 9925766; 8261423
31	Myocardial ischemia	31778269; 31741439; 28885393	28174168
32	Neoplasm metastasis	29181060; 19536148; 16699298; 15856028; 28331341	22681645; 25120023; 10952782; 12239456; 26252518; 16015609; 24705642; 18612712; 20581057; 23007696; 16157226
33	Neoplasms	19536148; 19544471; 29707043; 12902921; 15709172; 29345289; 26481148; 7982467; 18781955; 24467192; 19886790; 17429338; 21042786; 28871174; 11328280; 17992257; 30214643; 25182342; 30554729; 24059228; 17610479; 28418910; 19264915; 28714517; 30644531	22681645; 25120023; 10952782; 12239456; 26252518; 16015609; 24705642; 18612712; 20581057; 23007696; 16157226
34	Non-small cell lung carcinoma	31752873; 16909043; 27494133; 15709164; 29345289; 29132013; 30745811	18620780
35	Ovarian neoplasm	17893874; 17893875	19888321
36	Primary malignant neoplasm	31704854; 30483799; 31322257; 28795314; 15856028; 19536148; 25331796; 26412593;	19888321; 28123545; 29552203; 17970874

		30969847; 31596520; 18620752;16207704	
37	Primary malignant neoplasm of lung	29345289	31497188; 18620780
38	Prostate carcinoma	30958616; 29917167; 15467711	15280928
39	Squamous cell carcinoma	25182342; 15709164; 15967106; 16278379	17495951; 18201235; 29053662; 20596636; 18705642
40	Thyroid neoplasm	16699298	20629554
41	Tumor cell invasion	28247960; 22843933; 28629521; 25175278; 28795314; 15856028; 23028796; 24100272	24705642; 10952782; 29544367; 29629840
42	Tumor progression	23305401; 19886790; 28415825; 26481148; 29491103; 26412593; 18781955; 24497137; 12759237; 17922127; 26575329; 19544471; 17429338	22681645

Table S5. List of the human diseases associated with EPO and S100A6 protein, according to Open Targets Platform database (https://www.opentargets.org), and corresponding association scores (https://docs.targetvalidation.org/getting-started/scoring). Diseases with association scores for the both proteins exceeding 0.1 are highlighted in yellow.

№	*Disease*	*EPO*	*S100A6*
1	acanthosis nigricans	0.013	0.006
2	acrokeratoelastoidosis of Costa	0.004	0.002
3	acute coronary syndrome	0.015	0.009
4	acute hepatic failure	0.001	0.028
5	acute kidney injury	0.029	0.011
6	acute lymphoblastic leukemia	0.021	0.066
7	acute myocardial infarction	0.023	0.013
8	adenocarcinoma	0.008	0.023
9	adenoma	0.001	0.031
10	adrenal cortex carcinoma	0.018	0.025
11	adrenal gland pheochromocytoma	0.023	0.002
12	adrenocortical adenoma	0.001	0.001
13	alpers syndrome	0.005	0.003
14	Alzheimer's disease	0.097	0.005
15	amyotrophic lateral sclerosis	0.048	0.014
16	angina pectoris	0.009	0.002
17	anxiety	0.012	0.001
18	atrial fibrillation	0.021	0.003
19	atrophy	0.026	0.001
20	autoimmune disease	0.025	0.002
21	autonomic neuropathy	0.018	0.001
22	autosomal dominant polycystic kidney disease	0.084	0.002
23	axial length measurement	0.034	0.026
24	bacterial disease	0.017	0.002
25	Bartsocas-Papas syndrome	0.007	0.002
26	Bartter syndrome	0.008	0.004
27	benign prostatic hyperplasia	0.002	0.005
28	bladder carcinoma	0.012	0.009
29	brain injury	0.038	0.001
30	breast cancer	0.023	0.044
31	breast carcinoma	0.042	0.008
32	calf circumference measurement	0.040	0.009
33	cancer	0.084	0.043
34	carcinoma	0.011	0.017
35	cardiac hypertrophy	0.016	0.016
36	cardiomyopathy	0.015	0.001
37	cardiovascular disease	0.039	0.004
38	cervical carcinoma	0.012	0.002
39	cholangiocarcinoma	0.058	0.081
40	cholesteryl ester measurement	0.007	0.013
41	chromosomal aberration frequency	0.012	0.001
42	chronic kidney disease	0.116	0.005
43	chronic obstructive pulmonary disease	0.035	0.007

44	cirrhosis of liver	0.045	0.004
45	clear cell adenocarcinoma	0.004	0.004
46	clear cell renal carcinoma	0.001	0.022
47	cognitive disorder	0.002	0.001
48	colon carcinoma	0.037	0.003
49	colorectal adenocarcinoma	0.007	0.042
50	colorectal carcinoma	0.002	0.050
51	ongenital adrenal hyperplasia	0.008	0.003
52	congenital heart disease	0.014	0.001
53	congestive heart failure	0.087	0.004
54	coronary artery disease	0.040	0.001
55	Cushing syndrome	0.001	0.002
56	cyst	0.048	0.004
57	decubitus ulcer	0.004	0.007
58	diabetes mellitus	0.094	0.006
59	dilated cardiomyopathy	0.018	0.002
60	Down syndrome	0.021	0.001
61	dry eye syndrome	0.007	0.002
62	Duchenne muscular dystrophy	0.004	0.012
63	endometrial carcinoma	0.018	0.012
64	endometriosis	0.058	0.055
65	endometritis	0.007	0.002
66	fetal growth restriction	0.020	0.004
67	fibroma	0.004	0.002
68	Friedreich ataxia	0.025	0.001
69	gastric adenocarcinoma	0.013	0.003
70	gastric carcinoma	0.010	0.013
71	gastroenteritis	0.004	0.002
72	gastrointestinal stromal tumor	0.051	0.001
73	glaucoma	0.059	0.003
74	glioblastoma multiforme	0.026	0.016
75	heart disease	0.010	0.004
76	heart failure	0.024	0.004
77	hematocrit	0.326	0.002
78	hepatocellular carcinoma	0.090	0.054
79	hyperglycemia	0.082	0.006
80	hypertension	0.043	0.011
81	hypothyroidism	0.030	0.011
82	immature platelet fraction	0.003	0.004
83	infection	0.092	0.029
84	infertility	0.004	0.008
85	inflammatory bowel disease	0.037	0.001
86	influenza	0.011	0.001
87	intermediate coronary syndrome	0.002	0.007
88	invasive breast carcinoma	0.039	0.003
89	kidney disease	0.027	0.007
90	leiomyoma	0.032	0.010
91	leiomyosarcoma	0.018	0.001
92	leprosy	0.007	0.009

93	lung adenocarcinoma	0.009	0.008
94	lung carcinoma	0.008	0.004
95	lupus nephritis	0.010	0.002
96	lymph node metastatic carcinoma	0.007	0.012
97	malignant hypertension	0.012	0.004
98	meconium aspiration syndrome	0.007	0.002
99	medulloblastoma	0.004	0.024
100	melanoma	0.088	0.040
101	meningioma	0.011	0.016
102	meningococcal infection	0.004	0.002
103	mental deterioration	0.009	0.004
104	mesothelioma	0.003	0.004
105	metastatic malignant neoplasm	0.004	0.002
106	metastatic neoplasm	0.006	0.001
107	Miyoshi myopathy	0.084	0.025
108	monocytic leukemia	0.002	0.002
109	mucinous carcinoma	0.002	0.002
110	multiple myeloma	0.088	0.009
111	muscular dystrophy	0.003	0.001
112	myocardial infarction	0.035	0.011
113	myocardial ischemia	0.018	0.007
114	neoplasm	0.112	0.109
115	nephrotic syndrome	0.024	0.001
116	nervous system disease	0.025	0.008
117	neuroblastoma	0.034	0.020
118	non-alcoholic steatohepatitis	0.001	0.002
119	non-Hodgkins lymphoma	0.013	0.004
120	non-small cell lung carcinoma	0.093	0.031
121	nonpapillary renal cell carcinoma	0.040	0.079
122	obesity	0.087	0.004
123	obstructive sleep apnea	0.062	0.004
124	oral squamous cell carcinoma	0.001	0.007
125	osteoarthritis	0.011	0.001
126	osteosarcoma	0.052	0.026
127	ovarian carcinoma	0.013	0.028
128	ovarian dysfunction	0.004	0.003
129	Pallister-Hall syndrome	0.017	0.006
130	pancreas disease	0.009	0.003
131	pancreatic ductal adenocarcinoma	0.004	0.017
132	pancreatic neoplasm	0.019	0.006
133	pancreatitis	0.004	0.004
134	papillary thyroid carcinoma	0.010	0.008
135	papilloma	0.002	0.014
136	Patent ductus arteriosus	0.009	0.010
137	periodontitis	0.035	0.001
138	pes	0.039	0.050
139	Pick disease	0.002	0.001
140	pneumonia	0.005	0.002
141	polycystic ovary syndrome	0.015	0.009

142	portal hypertension	0.005	0.002
143	primary hypertension	0.026	0.003
144	protein measurement	0.086	0.180
145	pterygium	0.007	0.004
146	pulmonary fibrosis	0.003	0.009
147	pulmonary hypertension	0.015	0.004
148	relapsing-remitting multiple sclerosis	0.001	0.002
149	renal cell carcinoma	0.106	0.002
150	renal insufficiency	0.082	0.002
151	respiratory failure	0.003	0.004
152	rheumatoid arthritis	0.052	0.009
153	sarcoidosis	0.009	0.002
154	sarcoma	0.013	0.012
155	schizophrenia	0.030	0.002
156	sepsis	0.065	0.013
157	septic shock	0.012	0.007
158	Sjogren syndrome	0.004	0.003
159	small cell lung carcinoma	0.016	0.005
160	squamous cell carcinoma	0.013	0.009
161	squamous cell lung carcinoma	0.001	0.091
162	status epilepticus	0.016	0.008
163	systemic lupus erythematosus	0.040	0.004
164	systemic scleroderma	0.008	0.004
165	thymoma	0.020	0.003
166	transient ischemic attack	0.019	0.001
167	type I diabetes mellitus	0.032	0.004
168	type II diabetes mellitus	0.052	0.003
169	undifferentiated pleomorphic sarcoma	0.002	0.003
170	viral disease	0.006	0.004
171	vitamin D deficiency	0.009	0.004

Table S6. List of the human diseases associated with EPO and S100P protein, according to DisGeNET database (http://www.disgenet.org). PubMed identifiers of the references confirming the protein-disease associations are indicated.

№	*Disease*	*EPO*	*S100P*
1	Adenocarcinoma	15709164	15632002;18575778;18162774
2	Ovarian neoplasm	17893874; 17893875	17539915
3	Polycystic ovary syndrome	30292674	21411543
4	Prostatic neoplasms	29917167; 15467711	17448597; 18452169

Table S7. List of the human diseases associated with EPO and S100P protein, according to Open Targets Platform database (https://www.opentargets.org), and corresponding association scores (https://docs.targetvalidation.org/getting-started/scoring). Diseases with association scores for the both proteins exceeding 0.1 are highlighted in yellow.

№	*Disease*	*EPO*	*S100P*
1	acne	0.002	0.015
2	acute coronary syndrome	0.015	0.001
3	acute respiratory distress syndrome	0.022	0.001
4	adenocarcinoma	0.007	0.023
5	Alzheimer's disease	0.097	0.001
6	angina pectoris	0.008	0.002
7	ankylosing spondylitis	0.007	0.003
8	asthma	0.019	0.009
9	bacterial disease	0.016	0.001
10	benign prostatic hyperplasia	0.002	0.002
11	bladder carcinoma	0.011	0.017
12	breast cancer	0.022	0.020
13	breast carcinoma	0.042	0.035
14	calf circumference measurement	0.039	0.014
15	cancer	0.084	0.092
16	carcinoma	0.011	0.016
17	cervical carcinoma	0.011	0.014
18	cholangiocarcinoma	0.058	0.041
19	chronic kidney disease	0.115	0.031
20	chronic periodontitis	0.001	0.004
21	cirrhosis of liver	0.044	0.001
22	clear cell adenocarcinoma	0.004	0.011
23	colitis	0.033	0.009
24	colon adenocarcinoma	0.009	0.011
25	colon carcinoma	0.036	0.014
26	colorectal adenocarcinoma	0.007	0.011
27	colorectal cancer	0.010	0.014
28	colorectal carcinoma	0.002	0.078
29	corpuscular hemoglobin content	0.002	0.003
30	diabetes mellitus	0.093	0.004
31	endometrial carcinoma	0.017	0.078
32	familial adenomatous polyposis	0.011	0.003
33	gastric carcinoma	0.010	0.011
34	glioblastoma multiforme	0.026	0.074
35	hepatocellular carcinoma	0.090	0.093
36	infection	0.092	0.005
37	inflammatory bowel disease	0.037	0.012
38	influenza	0.011	0.003
39	kidney neoplasm	0.025	0.008

40	lung adenocarcinoma	0.008	0.022
41	lung carcinoma	0.007	0.002
42	lymph node metastatic carcinoma	0.006	0.006
43	maternal uniparental disomy of chromosome 20	0.001	0.004
44	mean platelet volume	0.034	0.036
45	medulloblastoma	0.004	0.004
46	melanoma	0.080	0.017
47	metastatic carcinoma	0.002	0.002
48	metastatic malignant neoplasm	0.003	0.003
49	metastatic neoplasm	0.005	0.002
50	mucocutaneous lymph node syndrome	0.004	0.001
51	myocardial infarction	0.035	0.011
52	neoplasm	0.112	0.108
53	neuroblastoma	0.034	0.002
54	oral squamous cell carcinoma	0.001	0.005
55	osteoarthritis	0.011	0.004
56	osteosarcoma	0.052	0.019
57	ovarian carcinoma	0.012	0.004
58	pancreas disease	0.008	0.001
59	pancreatic adenocarcinoma	0.016	0.023
60	pancreatic ductal adenocarcinoma	0.004	0.027
61	pancreatic neoplasm	0.019	0.073
62	pancreatitis	0.003	0.002
63	pancytopenia	0.012	0.001
64	papillary thyroid carcinoma	0.009	0.004
65	paraganglioma	0.007	0.004
66	periodontitis	0.035	0.002
67	peripheral neuropathy	0.008	0.008
68	pneumonia	0.004	0.005
69	psoriasis	0.001	0.019
70	psoriatic arthritis	0.001	0.019
71	pulmonary arterial hypertension	0.082	0.002
72	relapsing-remitting multiple sclerosis	0.001	0.002
73	renal cell carcinoma	0.105	0.006
74	respiratory system disease	0.004	0.007
75	rheumatoid arthritis	0.052	0.019
76	sarcoma	0.013	0.001
77	small cell carcinoma	0.002	0.014
78	squamous cell carcinoma	0.012	0.001
79	thrombocytopenia	0.016	0.001
80	triple-negative breast cancer	0.005	0.009
81	type I diabetes mellitus	0.032	0.006
82	type II diabetes mellitus	0.005	0.013
83	ulcerative colitis	0.013	0.024
84	viral disease	0.005	0.001

Table S8. List of the human diseases associated with EPO and S100A2, S100A6 and S100P proteins, according to Open Targets Platform database (https://www.opentargets.org), and corresponding association scores (https://docs.targetvalidation.org/getting-started/scoring).

№	*Disease*	*EPO*	*S100A2*	*S100A6*	*S100P*
1	adenocarcinoma	0.008	0.012	0.023	0.023
2	Alzheimer's disease	0.097	0.010	0.005	0.001
3	benign prostatic hyperplasia	0.002	0.027	0.005	0.002
4	bladder carcinoma	0.012	0.069	0.009	0.018
5	breast cancer	0.023	0.006	0.044	0.020
6	breast carcinoma	0.042	0.004	0.008	0.035
7	cancer	0.084	0.048	0.043	0.092
8	carcinoma	0.011	0.009	0.017	0.016
9	cervical carcinoma	0.012	0.002	0.002	0.014
10	cholangiocarcinoma	0.058	0.069	0.081	0.041
11	colorectal adenocarcinoma	0.007	0.035	0.042	0.010
12	diabetes mellitus	0.094	0.002	0.006	0.004
13	gastric carcinoma	0.010	0.023	0.013	0.012
14	glioblastoma multiforme	0.026	0.012	0.016	0.074
15	hepatocellular carcinoma	0.090	0.004	0.054	0.094
16	infection	0.092	0.003	0.029	0.005
17	lung adenocarcinoma	0.009	0.017	0.008	0.023
18	lung carcinoma	0.008	0.002	0.004	0.003
19	lymph node metastatic carcinoma	0.007	0.003	0.012	0.007
20	medulloblastoma	0.004	0.043	0.024	0.004
21	melanoma	0.088	0.030	0.040	0.017
22	metastatic malignant neoplasm	0.004	0.002	0.002	0.004
23	neoplasm	0.112	0.061	0.109	0.108
24	oral squamous cell carcinoma	0.001	0.004	0.007	0.005
25	osteosarcoma	0.052	0.003	0.026	0.019
26	ovarian carcinoma	0.013	0.009	0.028	0.004
27	pancreatic ductal adenocarcinoma	0.004	0.010	0.017	0.028
28	periodontitis	0.035	0.032	0.001	0.003
29	renal cell carcinoma	0.106	0.036	0.002	0.006
30	squamous cell carcinoma	0.013	0.020	0.009	0.003

[1] Pace, C.N., Vajdos, F., Fee, L., Grimsley, G. and Gray, T. (1995). How to measure and predict the molar absorption coefficient of a protein. Protein Sci. 4, 2411-2423.
